# Putting the ‘teachable moment’ in context: A view from critical health psychology

**DOI:** 10.1177/13591053221101750

**Published:** 2022-06-07

**Authors:** Abigail Locke

**Affiliations:** Keele University, UK

**Keywords:** alcohol, critical health psychology, parenting cultures, pregnancy, teachable moment

## Abstract

The concept of ‘Teachable Moment’ (TM) is an increasingly used term within mainstream health psychology in relation to interventions and health behaviour change. It refers to a naturally occurring health event where individuals may be motivated to change their behaviours from unhealthy ones to healthier choices. Pregnancy is seen as a key time for behaviour change interventions, partly due to the idea that the mother has increased motivations to protect her unborn child. This paper proposes a Critical Health Psychological (CHP) re-examination of the concept and explores the ‘teachable moment’ within a wider framing of contemporary parenting ideologies in order to offer a more critical, nuanced and contextual consideration of pregnancy and the transition to motherhood. The paper locates these discussions using an example of alcohol usage in pregnancy. In doing so, this paper is the first of its kind to consider the ‘teachable moment’ from a critical health psychological perspective.

## Introduction

The ‘teachable moment’ (TM) is an increasingly used term within mainstream health psychology in relation to health behaviour change. It has been used to describe naturally occurring health events and, in particular, to consider the ways in which individuals are motivated to change their behaviours and move from engaging in ‘risky’ and unhealthy behaviours to ones that are deemed healthier choices. The TM sits very much within a neoliberal framing of informed choice and risk. Within health research, there are a growing number of studies considering the impact of teachable moments in different contexts of health behaviours including sexual behaviours and HIV prevention (Fabiano, 1993), alcohol use interventions (Roy and Worsham, 2017), diet, exercise and obesity (Atkinson et al., 2016) and other general settings within health promotion and mainstream health psychology. The TM has often being used as a focus to tackle single ‘unhealthy’ behaviours, such as smoking or drinking alcohol, rather than as a programme of complex behaviour change.

Pregnancy is one particular area in which many of the ‘teachable moment’ studies have been conducted and attention has been paid to topics including smoking behaviours and weight management in pregnant women.^1^ Pregnancy is seen as a key time for behaviour change interventions partly due to the idea that the mother has increased motivations to protect her unborn child. This paper proposes a critical re-examination of the teachable moment, considering its placing and usage in mainstream health psychology including in behaviour change, before considering a more nuanced, critical health psychological reading of the teachable moment. A critical health psychology perspective lens brings in a wider context, including reproductive justice, contemporary parenting ideologies and parenting cultures. By applying a critical health psychology perspective, and using alcohol in pregnancy as an exemplar, this paper explores the teachable moment in this wider framing in order to offer a more critical, nuanced and contextual consideration of the health area and the potential interventions and success thereof. The framing of this paper is novel and is the first known discussion of applying a critical lens to this upcoming concept of behaviour change within health psychology.

### The concept of a ‘teachable moment’

What becomes apparent from a review of the TM literature is how it appears to be a term that is used colloquially in different places in health promotion and health psychology and, as yet, there is no explicit and consistent definition that can be located. This paper will begin by exploring the attempts to theorise the TM before mapping its usage across health promotion and mainstream health psychology. One of the first attempts to map and theorise the TM was from McBride et al. (2003) who considered the potential of the teachable moment in changing smoking behaviours. They considered the ‘teachable moment’ as a concept that has been used to describe naturally occurring health events and the ways in which individuals are motivated to change their behaviours, perhaps due to these life events, where individuals move away from ‘riskier’ behaviours (potentially related to diet and exercise for instance) to more ‘healthy’ behaviours and actions. According to McBride et al. (2003) the occurrence of teachable moments is supported by conceptual models that emphasise the importance of cues in promoting motivation for behaviour change (e.g. Hochbaum, 1958). However, they also noted that despite what, in principle, look like tangible benefits of the teachable moment for health promotion and outcomes, there is no evaluation to support their existence either at a broad or specific level. McBride et al reviewed all of the articles related to teachable moments and smoking. They found that from 160 publications, 3/4 of these were observational studies and in these articles the usage of a teachable moment fell into following event categories: office visits, reproductive health, smoking related acute and chronic care, notification of abnormal test results, pregnancy, and disease diagnosis. They found some differences in cessation rates per smoking context (e.g. those who were pregnant or became hospitalised reported lower smoking rates than others).

Drawing on the previous research in the teachable moment and the existing conceptual models of behaviour change (e.g. Health Belief Model, Rosenstock, 1966), McBride et al proposed a Teachable Moment heuristic that included three constructs that would underlie whether a cueing event was significant enough to be a TM for, in the case of their work, smoking cessation. These were: (1) perceptions of personal risk and outcome expectancies; (2) the affective response so the emotional or affective responses that will impact how someone feels and how they display their behaviours; (3) changes in social role and self concept. They suggested that not all three domains had to be present for a TM but would assist with the potency of what could encompass a TM. As McBride et al, noted a ‘large body of literature suggests that social expectations of roles and norms are more impactful when failure to comply results in social stigmatisation’ (p. 164). They concluded that teachable moments are promising as an area of research with regards to interventions and behaviour change models. However, they also concluded that TM research was in its infancy and more modelling was needed in order to operationalise the TM for this purpose.

As McBride et al.’s review made apparent, understanding what actually counts as a teachable moment is complex and ill-defined. Lawson and Flocke (2009) performed a concept analysis in an attempt to understand the teachable moment further. According to Lawson and Flocke (2009: 25) ‘teachable moments’ are events or circumstances which can lead individuals to positive behaviour change. However, from doing a database search across social science medical disciplines for teachable moment literature, they found that the teachable moment to have been poorly developed both conceptually and operationally. For them, the usage of the term of ‘teachable moment’ fell into three different categories. Firstly, the teachable moment was ‘synonymous with opportunity’ in 81% of cases with the focus on behaviour change. Secondly, a context that leads to higher than expected behaviour change is retrospectively as labelled a teachable moment in 17% of cases. Lastly, in 2% of cases, the teachable moment referred to a phenomenon that involved a cueing event that prompted specific cognitive and emotional responses. Despite Lawson and Flocke’s paper suggesting that in most cases the teachable moment was used in the same way as having an ‘opportunity’, they noted that this ‘opportunity’ is a somewhat unpredictable one. That is, whilst the teachable moment is considered as the opportune moment for instruction and or learning, without a more in depth understanding and consideration of the many factors that can influence what it is, how it occurs, and how best to develop interventions, the TM is likely to remain an under-theorised and under-operationalised concept. Many of the operationalisations of the TM have been developed through the framework of the Health Belief Model (as per the examples above). However, with studies of health behaviour change, there are newer and potentially more influential models. Olander et al. (2016) proposed moving past the existing models of TM and behaviour change – the ‘existing moment’ through using the Capability-Opportunity-Motivation Behaviour framework (the COM-B model) developed by Michie et al. (2011). The COM-B framework proposes that behaviour has three necessary determinants: capability, opportunity and motivation. Olander et al. argue that the sole focus on motivation from previous teachable moment research may not be sufficient. Instead, they proposed that applying the COM-B framework to the concept of the TM, with the addition that these two other aspects of capability and opportunity, provides a richer analysis of potential determinants of health behaviour change and opportunities for interventions to occur. They also argue that a lack of capability and opportunity may act as barriers to behaviour change that otherwise would be based solely on changes in motivation. Olander et al. (2016) applied this conceptualisation of the TM to pregnancy which will be discussed in the following section.

## Pregnancy as a teachable moment

Pregnancy has been referred to as an opportune teachable moment because of the presumption that the expectant mother has a strong motivation to protect the well-being of her child. Secondly, there is also a strong social pressure to avoid ‘risky’ health behaviours such as smoking and drinking alcohol during pregnancy. It was Phelan (2010) who first suggested that pregnancy could be a ‘powerful “teachable moment” for promoting’ healthy behaviours in pregnancy. Phelan focussed this behaviour change on excessive gestational weight gain and suggested that a combination of advice around healthy eating and physical exercise could play a key role in preventing obesity for these women in the future.

As part of McBride et al.’s (2003) review on the TM and smoking, they considered published studies considering pregnancy, smoking and the teachable moment. They noted that in the studies that they considered, many of the expectant mothers ceased smoking during their pregnancies, thereby noting that expectant mothers do adopt more healthy behaviours during pregnancy. However, it appeared that such behaviour changes were not permanent and in the year after pregnancy, many mothers started smoking again (e.g. Haug et al., 1994).

According to Olander et al. (2016) the teachable moment idea has been underutilised in maternal health research and their approach which suggested the COM-B model in their paper titled ‘beyond the ‘teachable moment’ a conceptual analysis or women’s perinatal behaviour change’, suggested that the TM may offer ‘opportune points for intervention both prenatally and indeed postnatally’. For Olander et al. (2016) even if pregnancy is a teachable moment based on changes and motivation, health professionals may not intervene at the most appropriate times and thus the attempt to change behaviours may face difficulties or fail. They argue that to maximise the potential of the teachable moment for behaviour change, we need to seize opportune intervention moments during and after pregnancy. However, as Phelan (2010) had noted previously in her discussions on pregnancy as a TM, the challenge remained on how to translate research findings into clinical practice. (c.f. Warin, 2015, on different epistemologies concerning health interventions). Rockliffe et al. (2022) mapped the COM-B and Teachable Moment models across a thematic synthesis of 92 studies (see Rockliffe et al., 2021) that reflected factors influencing antenatal health behaviours in order to see which model was better for understanding behaviour change in pregnancy. They argued that the COM-B model mapped the nine themes identified in their previous synthesis whereas the TM model failed to incorporate some of the themes. However, they noted that currently neither model was able to fully capture the context of pregnancy.

A great deal of focus on applying the concept of the ‘teachable moment’ as a method of behaviour change has been related to diet and physical activity in pregnancy, as in the case of Phelan (2010) and Olander et al. (2016) above. Dinsdale et al. (2016) conducted a qualitative thematic analysis using interviews with new mothers who had all been put on one of three maternal obesity care pathways. The interviews focussed on the mother’s experiences of weight management support during their pregnancies and considering the opportune timing of pregnancy for behaviour change. Dinsdale et al found that the mothers were open to such interventions during and after pregnancy. However, they did also note that good communication was key as well as tailored and individualised interventions. Finally, they suggested that the ‘teachable moment’ window needs to be extended to post-partum to continue opportunities for behaviour change. Similarly, Atkinson et al. (2016) interviewed women about their decision making behaviours for diet and physical activity during pregnancy. Performing an Interpretative Phenomenological Analysis (IPA) on interviews with seven women, they argued that pregnancy may provide a teachable moment for positive health behaviour change. However, given that much of the current work focuses on the opportunity to create behaviour change, Atkinson et al.’s findings suggest that this in itself is more complex and the women interviewed suggested that the majority of the behaviour change was actually automatic and they adopted a new lifestyle of ‘healthy behaviours’ immediately on discovering their pregnancy. According to the women, their behaviour was driven by anxiety and a drive to minimise potential risks to the pregnancy, in line with expected, and acceptable, behaviours during pregnancy. Atkinson et al. (2016) concluded that the influence of health professionals was less influential and therefore ‘pregnancy alone may not create a “teachable moment”’ (p. 842). Casting a wider lens to the topic of pregnancy, we can suggest that what is seen as the ‘teachable moment’ actually becomes enmeshed in the identity changes that occur during the transition to motherhood. Thus, the expectant mothers adopted healthier behaviours in their new mothering identity to protect their babies and fit into societal norms of ‘good mothering’ behaviours.

What the previous research studies have demonstrated is that the concept of the teachable moment is not altogether clear in either definition or delivery. Indeed it becomes more complex when we are considering the use of the TM in pregnancy because, as Atkinson et al. (2016) suggested, women were adopting behaviours because they were pregnant despite/unrelated to their interactions with health professionals. This suggests that by simply proposing an intervention to be given at a particular time (the ‘teachable moment’), health psychologists and health professionals may be missing crucial nuance and context in which women are making key decisions around health related behaviours that may impact both them and the foetus.

## A critical health psychology approach to the teachable moment

Many of the behaviour change models (e.g. Health Behaviour Change Model, COM-B) reside with what has been termed, by some, as ‘mainstream’ health psychology. Concepts such as the TM are typically drawn from social-cognitive models and biological psychology, as well as influence from diverse fields such as health economics, behavioural medicine and sometimes medical sociology. As critiques of this mainstream approach have documented, such an approach works from a positivist or post-positivist perspective and is criticised for being heavily individualistic and de-contextualised (Marks, 1996; Murray, 2015). In contrast, Critical Health Psychology (CHP) challenges assumptions and practices of the mainstream approach, arguing that people are ‘complex, changing and multifaceted’ (Chamberlain and Murray, 2017: 432–433). CHP considers how individuals can be empowered or disenfranchised through knowledge of health contexts and implements a wider contextual consideration to attempts to change health behaviours. In this sense then CHP integrates a wider socio-cultural view including, for example, structural inequalities and socio-economic factors in, considering the differences in health behaviours, norms and outcomes amongst different groups. When considering infant feeding practices, the ‘norm’ to formula feed amongst certain groupings is portrayed as a choice. As Murphy et al. (1998) note what we regard as ‘choices’ are rarely actual choices but rather embedded within wider material and social contexts:

“We are arguing that it is misleading to treat women’s feeding practices as the simple expression of individualistic preferences. Rather, such practices reflect the material and sociocultural contexts in which their decisions are made. The risk of defining such decisions as choice is that we camouflage the constraints under which women deliberate and act”. (Murphy et al., 1998: 262–263)

The attention that a CHP approach brings to the ways in which wider social-psychological, socio-political, bio-political and socio-cultural processes may influence the ‘choices’ that are available to us in relation to our health and wellbeing practices, gives a more integrated view of the interplay involved in understanding the factors that may have an influence on potentials for health behaviour change. What I am advocating in this paper is that by applying a CHP approach, coupled with the context of contemporary parenting ideologies, we begin to understand the complexity and nuance in considering health promotion and behaviour change interventions at times such as pregnancy. There is a second related lens that is also relevant here of reproductive justice. The ‘Reproductive justice’ approach (West, 2009) has been incorporated into a feminist health psychology (Macleod, 2012) as one ‘that highlights the contextual nature of women’s lives. Given overarching socioeconomic inequalities, racism and sexism that shape many women’s lives, the reproductive justice approach focuses on achieving conditions that are necessary for comprehensive reproductive and sexual freedom.’ (Macleod, 2012: 159). If we are to apply a reproductive justice lens in with our critical (and feminist) health psychology perspective, we can see how decisions and choices that are made by expectant mothers need to be considered within the context in which they are made, with a wider consideration of intersectional factors and identities that are shaping how these ‘choices’ and decisions are realised.

## Critical health psychology, parenting culture studies and contemporary parenting ideologies

As we saw in the larger discussion around putting the teachable moment in context, the wider backdrop that influences and impacts on decision making is both broad and complex. The CHP approach, in conjunction with a move to mapping parenting cultures and contemporary parenting ideologies, offers key insights into understanding messaging and receipt of health advice and promotion of healthy behaviours. Contemporary parenting cultures in the Global North at least, have been regarded as being ‘intensive’. In formative and ground-breaking work by Hays (1996) on the ‘Cultural contradictions of motherhood’, she introduced the concept of ‘intensive mothering’, whereby the ‘good mother’ is depicted as being self-sacrificial and child-centred in her parenting practices. A similar ethos with regards to motherhood was presented by French philosopher Badinter (2013) who referred to the current mothering ideologies and the turn to the natural discourse pervasive throughout much of it as ‘overzealous motherhood’. Badinter continued with the suggestion that women who do not or cannot live up to this idealised form of motherhood may feel judged as being ‘bad’ (or inadequate) mothers (see also Arendell, 2000; Christopher, 2012). Therefore, these ‘good mothering’ discourses permeate through parenting practices and health advice even in the transition to motherhood and mothering identities that occur in pregnancy. However, these constructions of ‘good motherhood’ run counter to the realities of many mothers’ lives (Phoenix and Woollett, 1991).

When considering contemporary parenting ideologies, it becomes apparent that parenting practices are under ‘surveillance’ (Gross and Pattison, 2006). That is, there is a public sanctioning of expected behaviours and actions during pregnancy. Gross and Pattison link the idea of surveillance to a ‘pregnancy as containment’ discourse whereby the expectant mother’s embodiment ‘is refocused on the body contained within them’ (p. 139) and their identity becomes or lost or changed as the focus moves to protecting the foetus. Similarly, Deborah Lupton in her work on risk (Lupton, 1999) highlights the web of surveillance that expectant mothers experience in terms of expected health behaviours to protect the foetus. As Marotta (2008) commented ‘the cultural processes of motherhood to shape identity of women to replace it with the identity of mother’ (p. 203). If we consider both health advice and actual health behaviours during pregnancy, I would argue that we need to do so within a consideration of both the societal discourses of adopting a ‘good mothering’ identity (Arendell, 2000) whilst in the transition to motherhood (Smith, 1999).

The dominant discourses that are present within current health promotion practices are of concepts such as ‘informed decision making’, ‘informed choice’ and ‘risk’. These discourses permeate through health advice and expected behaviours for ‘good citizens’. Within the field of Parenting Culture Studies (e.g. Lee et al., 2014), we can see how dominant discourses around parenting are located within risk behaviours and risk management, drawing on work from Furedi (2002) on ‘Paranoid Parenting’ in which he notes parenting has been reconstructed as a ‘troublesome enterprise. . .which systematically deskills mothers and fathers’ (p. 201). A key concept from Parenting Culture Studies is that of ‘parental determinism’ (Furedi, 2002; Lee, 2014) which means that the actions of the parent (including expectant parent) are considered in terms of effects on the child, as Ellie Lee notes:

“Parental action, in most areas of everyday life, is now considered to have a determining impact on children’s future happiness, healthiness and success”. (Lee, 2014: 2).

As has been claimed elsewhere, mothers are typically cast as ‘risk managers’ (Lee et al., 2010) in that their role is to make ‘informed choices’ on what the appropriate actions are in their parenting practice. Working from a neoliberal standpoint, there is a presumption here that we are citizens of a liberal democracy making choices about our lives and our health that are based on accurate and true information that we receive in order to avoid or minimise the risk of harm to ourselves or our families (Ayo, 2012). The way that parenting discourses have become bound up with notions of risk links in with Foucault’s notion of governmentality (Foucault, 1991; Lupton, 1999; O’Malley, 2008) and ‘technologies of the self’ (Foucault, 1988) whereby individuals become accountable for the management of social or health risks that they become involved in. In turn, many of these discourses become tied up with health behaviours related to parenting, and discourses around moral accountability and deviance. Furthermore, aspects of accountability (and potential blame) for making the ‘wrong’ choice (Phipps, 2014) are inherent throughout contemporary parenting discourses and start during pregnancy, even pre-conception as Waggonner (2017) claims in her work on the ‘zero trimester’.

## A case to demonstrate: Alcohol in pregnancy

To demonstrate the complexity of the TM and the need to put it in context, I will now consider alcohol in pregnancy as a case study as this is one that demonstrates how biomedical discourses of health promotion, and a reliance on the concept of ‘absolute risk’, may clash with the social/political/relational nature of decision making in pregnancy.

When we consider alcohol use in pregnancy, there is evidence of the negative effects of drinking in pregnancy and heavy alcohol consumption with a focus on strategies to target those at risk of problem drinking (e.g. Mengel et al., 2005; Scholin et al., 2019). However, the research evidence as to effects of low alcohol consumption in pregnancy is less clear and subject to much debate. For example, one epidemiological study of the effects of light drinking in pregnancy on children’s behavioural and cognitive abilities at 3 years of age found no evidence of any harmful effects (Kelly et al., 2009; see also Gray and Henderson, 2006; Henderson et al., 2007). Whilst other studies suggest that even one alcoholic drink a day in pregnancy may increase the risk of restricted growth (Hannigan and Armant, 2000). It is estimated that globally around 9.8% women consume alcohol during pregnancy, with about 14.6 per 10,000 people estimated to be affected by Foetal Alcohol Spectrum (Popova et al., 2017).

In the UK, Department of Health findings from the mid-200s were that 9% of women drank more than the recommended limit when pregnant (O’Brien, 2007). Some commentators have claimed that the only safe message for women during pregnancy is to abstain completely (e.g. Mukherjee et al., 2005) and this discourse appears to be gaining momentum at policy level. Different commentators have argued that this approach is ‘overly paternalistic’ (O’Brien, 2007) or ‘the only sensible message’ (Nathanson et al., 2007). Currently the advice given may be contradictory and research has suggested that advice around alcohol use in pregnancy should form part of all antenatal advice giving (Scholin et al., 2019). Women themselves seem to be unclear as to safe limits of alcohol in pregnancy, for example, in Raymond et al.’s (2009) study the mothers reported that most of them had drunk alcohol whilst pregnant and found the advice about safe levels of consumption both confusing and lacking in evidence. Given the widespread media coverage of these debates and issues, women are likely to have been exposed to widely varying and inconsistent messages about alcohol consumption (e.g. Lee et al., 2016). There has been little research on women’s own views and experiences when drinking in pregnancy, not least because of the ‘stigma’ associated with engaging in ‘risky’ behaviours whilst pregnant (c.f. Wigginton and Lee, 2013, on stigma and identity in pregnant women who smoke).

In addition to these inconclusive results there is also conflicting advice coming from different governments and healthcare bodies. For example, in the UK, the Department of Health changed its advice on alcohol consumption of no more than 2 units of alcohol per week to advising complete abstention (Department of Health, 2007). However, the National Collaborating Centre for Women’s and Children’s Health (2008) issued advice in March 2008 suggesting that women should drink a maximum of 1–2 units once or twice a week. Similar advice came from the Royal College of Obstetricians and Gynaecologists (RCOG, 2006) and in 2018, their advice was the following ‘Avoiding alcohol during pregnancy is the safest option. There is no proven safe amount of alcohol a woman can drink during pregnancy’. (RCOG, 2018: 1). This advice drew on the Chief Medical Officer’s report on alcohol use (Department of Health, 2016) that stated in terms of alcohol use in pregnancy that the ‘current evidence supports a “precautionary” approach and that the guidance should be clear that it is safest to avoid drinking alcohol in pregnancy’. (p. 8).

This advice was somewhat reflected elsewhere. For instance, in the UK, Public Health England’s (PHE, 2018) most recent guidelines on reproductive health and planning suggested a focus on health protective practices from those considering starting to plan to have a family. Similar discourses were seen around the globe, for example Centers for Disease Control and Prevention (CDC, 2016) in the USA suggested the avoidance of alcohol in those women who are of child-bearing age and not actively avoiding pregnancy. The rationale behind such an approach has been justified on the basis that the first 12 weeks of pregnancy are deemed critical in terms of development. Yet, it is in this period that many women do not realise that they pregnant and therefore may not be fully engaging in healthy practices, particularly if they had not been planning a pregnancy. The uncertainty in messages continued in the UK where in 2020, it was announced in the press as a suggestion on a draft policy from National Institute for Clinical Education (NICE) that alcohol intake during pregnancy should be noted on the child’s personalised child health record (PCHR) (National Institute for Health and Care Excellence [NICE], 2020). There has been much discussion on the legal and ethical grounds of this (e.g. Bennett and Bowden, 2022; Lee et al., 2022; Woollard, 2020) and the decision was overturned in March 2022. And in 2021, the World Health Organisation (WHO) made headlines for apparently suggesting in their draft Global Alcohol Action Plan (2022–2030) that women of child bearing age, whether planning pregnancies or not, are recommended to abstain for any alcohol intake due to a potential impact on any future pregnancies (WHO, 2021) (cf. Waggonner, 2017, ‘the zero trimester’). This was followed by headlines in newspapers such as the one below from The Independent in the United Kingdom:

“WHO CRITICISED BY PREGNANCY SERVICES FOR SUGGESTING ‘WOMEN OF CHILD-BEARING AGE SHOULD NOT DRINK ALCOHOL’

‘The WHO reduces women to little more than their reproductive capabilities’, charity says”

The Independent, UK, 17th June 2021

Headlines such as these have led some researchers to discuss how the issue of women drinking during pregnancy has become a ‘moral panic’ in health policies and promotion (Armstrong and Abel, 2000). Lowe and Lee (2010) discussed the abstinence policy brought in by the Department of Health (2007), contextualising it within a discussion of risk and Foetal Alcohol Spectrum Disorders (FASD), this despite the links between FASD and lower levels of alcohol consumption being inconclusive. Lowe and Lee address the neoliberal connotations of the choice/risk discourse and consider that whilst alcoholic consumption is constructed as a ‘choice’, it is one that is also constructed as off limits for pregnant women. They argue that:

“‘Playing safe’ by taking no risk, however small, has become ‘common sense’ when babies and children are involved (Furedi, 2002). It can be argued that abstinence policy reflects, in this light, ideas that are already ‘common sense’ in Britain about how parents should ‘parent’ their children and projects these assumptions backwards”. (Lowe and Lee, 2010: 308).

We can see Lowe and Lee’s viewpoint very much reflected in discourse around alcohol consumption and parenting with the backlash experienced by the World Health Organisation (WHO) for their recent suggestions around recommended alcohol consumption in women of child bearing age. Both CHP and a lens on parenting cultures fit within Foucault’s notions of governmentality and neoliberal constructions of informed choice and risk. Applying this to the case of alcohol in pregnancy, if a pregnant woman ‘chooses’ to drink during pregnancy, she becomes accountable for any ill effects on her pregnancy and unborn child (Ruhl, 1999).

Given the complexities inherent in the current example of alcohol and advice and pregnancy, it becomes apparent that the teachable moment as one example of an individualised, health behaviour change model needs would benefit from a more contextualised approach, particularly when addressing something as complex as occasional drinking during pregnancy. It is here that this paper proposes that a CHP a theoretical framework in which to offer an understanding of the TM in context. By applying a CHP perspective that draws on work around contemporary parenting ideologies and parenting culture studies, whilst also considering the experiences of pregnant women within the wider context of the transition to motherhood and maternal identities, we are able a deeper understanding of the complexities of delivering, and acting upon, advice to expectant mothers.

When we consider the information provided to women around drinking in pregnancy and the case for abstinence, it becomes apparent that this messaging is based on absolute risk discourses, despite the unclear evidence on light and moderate drinking on the foetus. This lack of clarity is apparent throughout much of the media and wider societal discourse around the issue. This uncertainty as to the ‘correct advice’ and ‘right actions’ in terms of which health behaviours to adopt may also be reflected to some extent in the discourse of parents and those who are either planning or expecting a child. Copelton (2008) considered the emotion work involved in managing ‘good mothering’ identities in expectant mothers drinking alcohol during pregnancy and the ways in which mothers either justified or excused their drinking behaviours. She claimed that:

“pregnant women do not respond passively to expert advice on pregnancy, nor do they drink in ignorance of the possible negative effects drinking might have” (Copelton, 2008:21–22).

Consider, for example, the post below from Mumsnet *Talk* which is an open access discussion forum hosted on Mumsnet where participant post different threads on topics of interest and concern. The following post is from a thread where a pregnant woman asks others on the site for their actions around alcohol in pregnancy and is reflective of Copelton’s (2008) findings.

## Mumsnet Talk – discussion on alcohol in pregnancy: Starter post

*“I know the official guidance is that zero alcohol is the safest option but there hasn’t been research on small amount of alcohol in pregnancy and apparently 80% of women drink something whilst pregnant.*

*I’m not looking for judgment here but in both of my pregnancies I’ve drunk a very small amount- like one small glass of wine per week sipped or a few sips on husbands glass.*

*What do others do and how much- be honest!”*

This post demonstrates that whilst the advice around drinking in pregnancy is known and understood, at least for the poster here, that ‘zero alcohol is the safest option’. However, she also notes the lack of clarity around light alcohol use in pregnancy and claims that around 80% of women have consumed alcohol whilst pregnant before noting her occasional use and asking site users for their experiences. The poster constructs themselves as reasonable and measured in their health behaviours, that is that whilst they have drunk through pregnancies, it is minimised as an ‘occasional’, ‘small’ drink and ‘sips’ instead of the heavy, binge drinking typically constructed with negative outcomes in pregnancy. In this brief example, we can see the poster managing two different discourses of ‘risk’ and ‘good mothering’. If we bring this back to the need to put the teachable moment and the potential for interventions into a CHP framed ‘context’, one that brings in wider parenting ideologies, we can see that the practice of being pregnant in society is infinitely more complex than behaviour change interventions can currently theorise. In this particular case, the expectant mother outlines both the official advice, the lack of evidence on light use, and finally her experience of alcohol usage in her pregnancies. As becomes evident from her post, she expects many other women to be partaking in the same behaviours as her (‘be honest’). By simply offering advice and behaviour change interventions around neoliberal framings of informed choice and risk without considering the complexities and context of people’s lives, the TM may somewhat miss its moment to create meaningful behaviour change.

The responses to her post seemed to follow a different number of tacks which reflect the uncertainty in these debates around risk discourses. The first was of abstinence with experiential messages that they drunk no alcohol during pregnancy. The second was closely related to the first and took the form of instructive messaging of total abstinence but contextualising it within a discourse of risk to the damaging foetus. The final tack was around experiences of moderate drinking during pregnancy with no risks and no blame.

The brief thread of messages on Mumsnet demonstrates a number of points that are relevant to this paper. The first is that it demonstrates that there is a difference between official health advice around alcohol use in pregnancy and the reported behaviours that expectant mothers are engaging in. We can suggest that some of this reflects the uncertainty around discourses of risk, health messaging, changing advice and a lack of clear evidence on moderate alcohol use in pregnancy, but there may be something else at play here too. Some of this may be explained through the identity transitions during pregnancy and motherhood, others as an almost resistance to the official advice and the ‘needs’ of the woman as herself and not simply as an (expectant) mother. This short excerpt also reflects how different discourses around alcohol use in pregnancy are being reflected in mundane everyday discourse, for example, total risk and abstinence arguments against moderation and ‘common sense’. Finally, it demonstrates the need to consider the complexities and context in which health decisions (about behaviours) are being made.

## Concluding thoughts

The ‘teachable moment’ is a concept that is increasingly coming into theories of behaviour change and health interventions. It has been applied to a variety of areas, one of which is considering the use of the TM as a behaviour change intervention in pregnancy and the early post-natal period. However, conceptualisations of the TM have been varied and, at times, poorly defined. In addition, concerns around decontextualised content and a focus on the individual that can arguably plague models of behaviour change in mainstream health psychology are relevant here in considering both the application and success of the TM. Therefore, this paper proposed a CHP perspective to the TM and sought to locate the teachable moment ‘in context’, using alcohol use in pregnancy as an exemplar. It did so in order to understand the wider issues that may need to be considered when attempting to operationalise the TM for a behaviour change intervention. Taking the growing literature on the TM in pregnancy, this manuscript contextualised the concept by locating it within contemporary parenting ideologies that draw on wider issues within social and political contexts. This may enable a clear understanding of what may be impacting on the choices and decisions that people make around their health and, in the case of parenting and the TM, the health of their families. With regards to the example of alcohol use in pregnancy, the wider contextual analysis demonstrated the uncertainty of the evidence on the risks of light to moderate alcohol use in pregnancy coupled with the changes in health advice to pregnant women which in turn led to some uncertainty on both ‘realistic’ and ‘appropriate’ health behaviours. All of this is set against a backdrop of ‘good mothering’ discourses and contemporary parenting ideologies. By adopting a CHP approach, which deals with both complexity and nuance of health ‘choices’ whilst locating the TM within this wider context, I argue that it enables us to offer a more in-depth understanding of what is actually occurring at particular moments in time, with the overall aim of being able to fully appreciate any barriers to behaviour change and consider the design of any interventions accordingly.

## Notes

1. While I do refer to pregnant ‘women’ throughout this paper, I recognise that it is important to acknowledge that not all pregnant people identify as women.
